# Revolutionizing Women’s Health: A Comprehensive Review of Artificial Intelligence Advancements in Gynecology

**DOI:** 10.3390/jcm13041061

**Published:** 2024-02-13

**Authors:** Marta Brandão, Francisco Mendes, Miguel Martins, Pedro Cardoso, Guilherme Macedo, Teresa Mascarenhas, Miguel Mascarenhas Saraiva

**Affiliations:** 1 Faculty of Medicine, University of Porto, Alameda Professor Hernâni Monteiro, 4200-427 Porto, Portugal; martasofiabrandao@gmail.com (M.B.); pedromarilio@gmail.com (P.C.); guilhermemacedo59@gmail.com (G.M.); tqc@sapo.pt (T.M.); 2 Department of Gastroenterology, São João University Hospital, Alameda Professor Hernâni Monteiro, 4200-427 Porto, Portugal; francisco.cnm@gmail.com (F.M.); miguel.pedro96@gmail.com (M.M.); 3 WGO Gastroenterology and Hepatology Training Center, 4200-427 Porto, Portugal; 4 Department of Obstetrics and Gynecology, São João University Hospital, Alameda Professor Hernâni Monteiro, 4200-427 Porto, Portugal

**Keywords:** artificial intelligence, gynecology, deep learning, machine learning

## Abstract

Artificial intelligence has yielded remarkably promising results in several medical fields, namely those with a strong imaging component. Gynecology relies heavily on imaging since it offers useful visual data on the female reproductive system, leading to a deeper understanding of pathophysiological concepts. The applicability of artificial intelligence technologies has not been as noticeable in gynecologic imaging as in other medical fields so far. However, due to growing interest in this area, some studies have been performed with exciting results. From urogynecology to oncology, artificial intelligence algorithms, particularly machine learning and deep learning, have shown huge potential to revolutionize the overall healthcare experience for women’s reproductive health. In this review, we aim to establish the current status of AI in gynecology, the upcoming developments in this area, and discuss the challenges facing its clinical implementation, namely the technological and ethical concerns for technology development, implementation, and accountability.

## 1. Introduction

Over the past years, interest and research in artificial intelligence (AI) technologies and their applicability to medical practice has considerably increased [1]. AI-based systems have made their way into a range of different medical fields, especially in those with a strong imaging component [2], offering exciting prospects for more efficient and effective use of medical images [3].

Artificial intelligence refers to a digitalized computer system that replicates the processing of the human brain [4], its intelligent behavior and critical thinking [5]. By using computer technology, these complex models have the potential to improve patient care by speeding up processes and increasing their accuracy and efficiency [6], with lower human demand [7]. It has proven its benefits in disease diagnosis and treatment, health management, drug research and development, and precision medicine [8].

Indeed, the world is facing a quickly evolving new era with growing needs for higher quality global healthcare [9]. As medical activity generates ever-increasing amounts of digital images and medical records, AI algorithms appear as candidates to handle these data efficiently.

When discussing the concept of artificial intelligence and its subsets, it is important to clarify that AI, machine learning (ML), and deep learning (DL) are overlapping disciplines [10]. In fact, ML uses computer algorithms automatically developed from input training data to recognize patterns within large databases [11]. Thus, these models appear as highly effective tools to predict future outcomes based on new unforeseen data and decision making in various disciplines [12]. Additionally, the models can be refined as new data are continuously added [13].

Furthermore, ML techniques can be either supervised or unsupervised [14]. A supervised algorithm uses a dataset that contains input features, such as output target pairs, labeled at the start of training, to learn mapping and establish meaningful relationships between the input data and the corresponding output, and creating a model that is able to differentiate among output labels. Then, the trained model takes in new, fresh, unseen data and makes predictions or classifications based on the knowledge from labeled examples [15]. Thus, these models depend heavily on high-quality labeled data. Moreover, once a model has been developed, it is tested on the new patient’s data, apart from those included in the training data, to determine its applicability to other people or scenarios [16].

On the other hand, unsupervised ML models are data-driven systems that automatically learn from the relationships between elementary bits of information associated with each variable of a dataset. Contrary to supervised ML, unsupervised ML methods reveal associations or clusters existing within datasets and model patterns without any predefined output data [17]. Unsupervised learning can be particularly beneficial and complement supervised ML approaches. As these methods can discover potentially unrecognized patterns from large databases, they can feed into supervised algorithms, which in turn will build new models to discriminate among the classes of interest [18].

Alternatively, DL is a subset of ML [11]. Convolutional neural networks (CNNs) are a complex multilayer architecture inspired by biological processes, since their design intends to replicate the structure and organization of the visual cortex, where interconnected neurons process and transmit information [19]. Therefore, they are particularly tailored to visual-imagery-related tasks.

Thus, AI algorithms, namely DL and CNNs, hold great promise in the field of medical imaging [2], from image recognition, processing, and reconstruction to automated analysis and classification [20]. Therefore, they are of great contribution to disciplines that rely heavily on images, and gynecology could be a player at the forefront in the development and application of AI models [21,22]. Table 1 succinctly explains the different ML and DL models characterized throughout the review.

## 2. Application in Gynecological Imaging

AI application in gynecology is still at an early stage when compared with other specialties. In fact, despite gynecology being one of the areas with the largest imaging component, the impact of AI in practice is still in an embryonic phase. Nevertheless, there is a need to understand the limitations of the available clinical imaging methods, namely clinician workload and intra and interobserver variability, and AI has the potential to overcome these limitations while increasing diagnostic accuracy [23]. However, AI has a huge and recognized potential to assist in repetitive tasks, such as automatically identifying good-quality images and identifying imaging patterns [21]. This work is a state-of-the-art review of AI advancements in gynecology.

### 2.1. Cervical Cancer

Cervical cancer is highly prevalent, with a cumulative worldwide incidence of 13.3 cases per 100,000 women-years, which is increased in low-income countries [24]. Additionally, it is associated with a mortality rate of 7.2 deaths per 100,000 women-years [24]. Furthermore, cervical cancer can be easily treated if detected at its early stages [25]. In daily practice, cervical cancer screening is based on human papillomavirus (HPV) testing and cytological examination. Therefore, it depends heavily on the pathologist’s experience, which also is less accurate and has high interobserver variability. Colposcopy is also a critical component of cervical cancer detection. However, because of the increased workload, visual screening leads to misdiagnosis and low diagnostic accuracy [26]. Several authors have advocated the potential of AI-powered cytological examination and colposcopy image analysis, identifying abnormal cells or lesions, thus strengthening cervical cancer screening and diagnostics [27]. This see-and-treat approach allows for earlier and effective treatment of lesions using minimally invasive procedures, such as thermocoagulation, reducing the malignancy and associated mortality [26], while reducing the need for unnecessary biopsies. Table 2 summarizes the most recent evidence about AI models in colposcopy.

The first to study the implementation of an AI model in cervical cancer diagnosis was Mehlhorn and colleagues, namely during colposcopy exams. In 2012, the group developed a computer-assisted diagnostic (CAD) device based on image-processing methods to automatically analyze colposcopy images. The CAD system revealed a diagnostic accuracy of 80%, with a sensitivity of 85% and a specificity of 75%, in differentiating normal or cervical intraepithelial neoplasia grade 1 (CIN1) from high-grade squamous intraepithelial lesions (HSILs)(CIN2 or CIN3) in colposcopy exams [28]. A second study by the same group confirmed the benefit of the CAD application during colposcopy exams’ evaluation, demonstrating an increase in diagnostic accuracy when the exam was evaluated by a less-experienced gynecologist [29]. A Greek group developed and trained a clinical-decision support system (CDSS) based on an artificial neural network to correctly triage 740 women before referral to colposcopy; this was based on the cytological diagnosis and the expression of various biomarkers [30]. Women detected with cervical intraepithelial neoplasia grade 2 or worse (CIN2+) were chosen to undergo colposcopy. The CDSS presented a sensitivity of 89.4%, a specificity of 97.1%, a positive predictive value of 89.4%, and a negative predictive value of 97.1%. This system has the potential to reduce the referral rate for colposcopy when applicated in clinical practice.

Sato et al. were the first to develop a preliminary DL model based on a Keras neural network with 485 images from 158 individuals who underwent colposcopy [31]. The CNN tried to classify colposcopy images and predict post-procedure diagnoses. Patients were classified into three groups: severe dysplasia, carcinoma in situ (CIS), and invasive cancer (IC). Rather than evaluating the performance of a given AI-based model itself, the authors wanted to establish its feasibility and usefulness in clinical practice as quick and efficient way to obtain an accurate preoperative diagnosis that could help doctors in the decision-making process. The model reached 50% accuracy in this dataset.

Asiedu et al. extracted color and textural-based features from visual inspection with acetic acid and lugol’s iodine, and then used the data to train a support vector machine (SVM) model to distinguish cervical intraepithelial neoplasia (CIN) from normal and benign tissue [32]. The proposed framework achieved a sensitivity, specificity, and accuracy of 81.3%, 78.6%, and 80.0%, respectively, achieving better performance than expert physicians using the same dataset. In the same year, Miyagi et al. developed a CNN for classification of cervical squamous intraepithelial lesions from colposcopy images of 330 patients, 97 with low-grade squamous intraepithelial lesions (LSILs) and 213 with HSILs, who underwent colposcopy and lesion biopsy [33]. The CNN differentiated HSILs from LSILs with higher accuracy (82.3% vs. 79.7%) and specificity (88.2% vs. 77.3%), although with slightly lower sensitivity (80.0% vs. 83.1%). A study by the same group in 2020 included the results of human papilloma virus (HPV) testing [34]. The trained CNN revealed an accuracy of 94.1%, higher than gynecologists’ 84.3% global accuracy. This study was one of the first to include additional variables in order to increase the diagnostic accuracy of the CNN.

In 2020, Yuan and colleagues worked on a database composed of 22,330 cases, including 10,365 normal cases, 6357 LSIL cases, and 5608 HSIL cases [35]. Based on a dataset of three frames per case, they developed a ResNet CNN for differentiating between normal images and dysplastic lesions (LSILs or HSILs). The CNN revealed 85% sensitivity, 82% specificity, and 93% accuracy. Also, they created a U-Net CNN capable of delimitating squamous lesions (LSILs or HSILs) in acetic acid and iodine images. The model had 84.7% sensitivity in acetic acid images and 61.6% in lugol’s iodine images. These lesion delimitation models are of utmost importance for guiding colposcopy-based biopsies. Finally, the group developed a MASK-R CNN model to detect HSILs. The model detected HSILs with 84.7% sensitivity in both acetic acid and iodine images, accurately identifying lesions that benefit from treatment.

A Chinese group carried out a study to develop and validate a Colposcopic Artificial Intelligence Auxiliary Diagnostic System (CAIADS) using digital records of 19,435 patients, including colposcopy images and pathological results, which was considered the gold standard [36]. Agreement between CAIADS-graded colposcopy and pathology findings was higher than in expert-interpreted colposcopy (82.2% vs. 65.9%). The CAIADS model was able to increase its diagnostic accuracy after considering patients’ related factors (such as previous cytology results). The new model also revealed a superior ability to predict biopsy sites, with a median mean-intersection-over-union (mIoU) of 0.758.

In 2021, Fu et al. intended to create a model incorporating the results of HPV typing, cytological examination, and colposcopy analysis [37]. First of all, they acquired colposcopy images and created a multiple-image-based DL model using a multivariable logistic regression (MLR), presenting an area under the curve (AUC) of 0.845. Then, the results of the cytology test and HPV test were used to build an ML model, with an AUC of 0.837. Finally, they built a cross-modal integrated model using ML, through combining the multiple-image-based DL model and the Cytology–HPV joint diagnostic model. The authors proved the synergetic benefits of the ensembled model, presenting a higher AUC of 0.921. A ShuffleNet-based cervical precancerous lesion classification method based on colposcopy images was developed by Fang and colleagues [38]. The image dataset was classified into five categories, namely normal, cervical cancer, LSILs (CIN1), HSILs (CIN2/CIN3), and cervical neoplasm. In this dataset, the colposcopy images were expanded to reduce the impact of uneven distribution between the lesions’ categories, Additionally, the ShuttleNet network was compared with other CNNs (like the RestNet or the DenseNet). The new CNN model presented a global accuracy of 81.23%, with an AUC of 0.99. A recent study by Chen et al. collected images from 6002 colposcopy examinations of normal cervixes and those with LSILs and HSILs [39]. A new model based on EficcientNet-B0 using Gate Recurrent Unit was developed in order to accurately identify HSILs. The CNN revealed a sensitivity of 93.6%, specificity of 87.6%, and accuracy of 90.6% in distinguishing between HSILs, LSILs, and normal-cervix images.

Additionally, the diagnosis of cervical cancer can also be guided using magnetic resonance imaging (MRI). Urushibara et al. designed a study including 418 patients, 177 patients with pathologically confirmed cervical cancer and 241 patients without cancer, who underwent MRI between 2013 and 2020 [40]. They compared the performance of a DL architecture, called Xception, with experienced radiologists in the diagnosis of cervical cancer on sagittal T2-weighted images. The CNN presented higher sensitivity (88.3% vs. 78.3–86.7%) and accuracy (90.8% vs. 86.7–89.2%), with similar specificity.

**Table 2:** Summary of Studies about AI implementation in colposcopy. Sn, sensititivy; Sp, specificity; AUC, area under the curve; CIN, cervical intraepithelial neoplasia; HSIL, high-grade squamous intraepithelial neoplasia; LSIL, low-grade squamous intraepithelial neoplasia; N, normal; VIA, visual inspection with acetic acid; VILI, visual inspection lugol iodine. NK—not known.

Author, Year	Study Aim	Patients *n*	Frames *n*	Pathologic Confirmation	AI Methoad	Dataset Method	Analysis Method	Catego-Ries	Performance Metrics %		
									Sn	Sp	AUC
Mehlhorn, 2012, Germany [28]	Detection of CIN 2/3 lesions	198	375 frames (VIA) – Normal: 39 – CIN 1: 41 – CIN 2: 99 – CIN 3: 19	Yes	Color texture analysis methods	frame annotation in VIA (normal vs. CIN I vs. CIN II-III)	*n*-fold cross validation	HSIL (CIN 2 or CIN3)	85	75	80
Asiedu, 2019, USA [32]	Differentiating normal vs. abnormal (CIN+)	134	Not known Only number of patients per category	Yes	SVM	frame annotiation in VIA and VILI (VILI/VIA positive vs. VILI/VIA negative)	5-fold cross validation (80–20%)	Abnormal (LSIL or HSIL)	81	79	80
Miyagi, 2019, Japan [33]	Differentiating LSIL vs. HSIL	330	1 frame per colposcopy (VIA) – LSIL: 97 – HSIL: 213	Yes	ResNet	frame labeling in acid free (LSIL vs. HSIL)	5-fold cross validation	LSIL vs. HSIL	80	88	83
Yuan, 2020, China [35]	Differentiating normal vs. abnormal (LSIL+)	22,330	3 frames per colposcopy (AF, VIA and VILI) – Normal: 10,365 × 3 – LSIL: 6357 × 3 – HSIL: 5608 × 3	Yes	ResNet	frame annotation in acid-free, VIA and VILI (normal vs. LSIL vs. HSIL)	Train–test validation (80–10–10%)	Abnormal (LSIL or HSIL)	85	82	93
	Predicting the area of lesion (LSIL+)	11,198	11,198 VIA frames + 11,198 VILI frames – Normal: NK – LSIL: NK – HSIL: NK	Yes	U-Net			VIA	85	NK	NK
								VILI	62	NK	NK
	Detection of HSIL	11,198		Yes	MASK R			VIA	85	NK	NK
								VILI	85	NK	NK
Xue, 2020, China [36]	Differentiating normal vs. LSIL vs. HSIL vs. cancer	19,435	101,7267 acid-free frames – Normal: NK – LSIL: NK – HSIL: NK – Cancer: NK	Yes	U-Net + YOLO	frame annotation in acid-free (normal vs. LSIL vs. HSIL vs. Cancer)	Train–test validation (70–10–20%)	LSIL+	87	49	69
								HSIL+	66	90	78
Chen, 2022, China [39]	Differentiating LSIL vs. HSIL	6002	18,006 frames (AF, VIA and VILI)	Yes	E-B0 with GRU	frame labeling in acid-free, VIA and VILI (LSIL vs. HSIL)	Train–test validation (60–20–20%)	LSIL vs. HSIL	88	94	91
Fang, 2022, China [38]	Differentiating normal vs. cervical cancer vs. LSIL vs. HSIL vs. cervical neoplasm	1189	6996 acid-free frames – Normal: 2352 – LSIL: 780 – HSIL: 2532 – Cervical cancer: 408 – Cervical neoplasm: 924	Not mentioned	ShuffleNet	frame labeling in acid free (normal vs. LSIL vs. HSIL vs. cervical cancer vs. cervical neoplasm) + data augmentation	train–test (90–10%)	N vs. all	90	NK	NK
								LSIL vs. all	86	NK	NK
								HSIL vs. all	82	NK	NK
								Cervical neoplasm		NK	NK
								Cervical cancer		NK	NK

The development of AI models in cervical cancer diagnosis can also be accomplished at the histological level. In fact, in 2019, Sompawong and colleagues applied a Mask Regional Convolutional Neural Network (Mask R-CNN) to analyze cervical cells using liquid-based histological slides and screening for abnormal nuclear features [41]. The proposed algorithm achieved an accuracy of 91.7%, sensitivity of 91.7%, and specificity of 91.7%. In the same year, a group of Indian pathologists trained a CNN to identify abnormal features from liquid-based cytology (LBCC) smears, using 2816 images—816 presenting abnormal features, indicating LSILs or HSILs, and 2000 normal images, containing benign epithelial cells and reactive changes [42]. The referred model yielded a sensitivity of 95.6%, with 79.8% specificity. In addition, its high negative predictive value of 99.1% makes it a potentially valuable tool for cervical cancer screening. The technological development was accompanied by a multicenter observational study that evaluated the performance of AI-assisted cytology for the detection of CIN or cancer [43]. The group used 188,542 digital cytological images to train a supervised DL algorithm. The DL model detected 92.6% of CIN 2 and 96.1% of CIN 3, showing an equivalent sensitivity but higher specificity compared to skilled senior cytologists.

In fact, a validated AI-assisted cytology system, called Landing CytoScanner^®^, was enrolled in a cohort study including 0.7 million women [44]. Women with abnormal results in both AI-assisted and manual readings were diagnosed using colposcopy and biopsy. The outcomes were of histologically confirmed CIN of grade 2 or worse (CIN2+). The agreement rate between AI and the manual reading was 94.7% and the kappa value was 0.92. The large number of images analyzed contributed to the robustness of this experiment. Given its ability to exclude most normal cytology, with increased sensitivity compared with manual cytology readings, the results support the AI-based cytology system for primary screening of cervical cancer in a large-scale population. More recently, a Chinese group studied the diagnostic performance of an artificial intelligence-enabled liquid-based cytology (AI-LBC) in triaging women with HPV [45]. AI-LBC achieved sensitivity for the detection of CIN2+ comparable to that of experienced cytologists (86.49% vs. 83.78%), but significantly higher in specificity (51.33% vs. 40.93%). Similar results were observed for CIN3+. Moreover, the AI-LBC reduced colposcopy referral by 10%, compared with cytologists, making the process more effective by reducing the number of false positives in the cytological evaluation. Even though there are positive conclusions, prospective designs are needed to test the triaging performance of the developed model.

In order to increase the diagnostic accuracy of cervical lesions, new image methods have been evaluated. High-resolution endomicroscopy (HRME) consists of a fiber optic fluorescence microscope capable of acquiring nuclear images in vivo. In 2022, Brenes et al. used a dataset of images from over 1600 patients to train, validate, and test a CNN algorithm to diagnose CIN2+ cases from HRME images [46]. The proposed method consistently outperformed the current gold-standard methods, achieving an accuracy of 87%, with a sensitivity of 94% and specificity of 58%. By incorporating the HPV status, specificity increased to 71%.

Finally, AI-models can also provide prognostic information, guiding therapeutic decision. In 2019, Matsuo et al. compared the performance of a DL model with four survival-analysis models, including the Cox proportional hazard regression model, the mainstay for survival analyses in oncologic research in predicting survival in women with cervical cancer [47]. The study included 768 women, with a median follow-up time of 40.2 months. The new model exhibited superior performance, outperforming the prediction models for overall survival, but with similar results in predicting progression-free survival. The prognostic information given using DL algorithms was also evaluated in a retrospective study evaluating 157 women who developed recurrent cervical cancer among 431 women with cervical cancer diagnosed between January 2008 and December 2014 [48]. Predictions of 3- and 6-month survival after recurrence were compared between the current approach (linear regression model) and their experimental approach (DL neural network model). The DL model inputs included some clinical and laboratorial parameters and achieved significantly better prediction for 3-month (AUC 0.747 vs. 0.652) and 6- month (AUC 0.724 vs. 0.685) survival. Better predictions of limited life expectancy in women with recurrent cervical cancer pave the way for even more personalized clinical decisions, thus helping clinicians to individually adjust the level of care provided.

### 2.2. Endometrial Cancer

Endometrial cancer is the most common gynecological malignancy in developed countries, with rising prevalence. Commonly, the disease is diagnosed in an early localized phase in the setting of postmenopausal bleeding. Nevertheless, cases with advanced disease at diagnosis have a poor prognosis [49]. Additionally, endometrial cytology is not a cost-effective screening method, with a large number of false negatives. In this context, AI algorithms represent a profitable tool either in the automatic classification of hysteroscopy or histopathological images necessary for diagnosing endometrial cancer, or in preoperative MRI-based predictions. Table 3 summarizes the main works about artificial intelligence models for the diagnosis of endometrial cancer during hysteroscopy.

Neofytou and colleagues were the first to develop a CAD system for the classification of hysteroscopy images based on color–texture analysis [50]. In total, 418 regions of interest were extracted from 40 patients, and these data were used to train two classifiers: a probabilistic neural network (PNN) and an SVM model. The latter achieved the highest percentage of correct classifications between normal and abnormal endometrial tissue (79%). In 2013, Vlachokosta et al. developed a neural network for the classification of hysteroscopic images of the endometrium by evaluating the endometrial vessels and texture features [51]. In this work, a Fuzzy C-Means clustering algorithm was used for feature selection. A total of 28 patients with abnormal uterine bleeding, 10 patients with endometrial cancer, and 39 subjects with no pathological condition were enrolled in the study. The neural network had an accuracy of 91.2%, with a sensitivity of 93.6%, and a specificity of 83.8%. The role of AI models in hysteroscopy was also addressed by Zhang et al. in 2021. The Chinese group obtained 1851 hysteroscopic images from 454 patients with confirmed endometrial lesions, including endometrial hyperplasia without atypia, atypical hyperplasia, endometrial cancer, endometrial polyps, and submucous myomas, to construct and train a VGGNet-16 model, a 16-layer DL CNN [52]. The model achieved an overall accuracy in classifying endometrial lesions of 80.8%. For dichotomous classification of the lesions as benign or as premalignant/malignant, the model’s accuracy, sensitivity, and specificity were 90.8%, 83.0%, and 96.0%, respectively. In both classification tasks the CNN model outperformed the gynecologist’s evaluation. A Japanese study developed a DL-based model with 411,800 images from 177 videos (comprising normal findings, endometrial polyps, endometrial myomas, atypical endometrial neoplasia, and endometrial cancer) [53]. The developed CCN had a binary nature (malign vs. benign or normal findings). Three different models were evaluated—Xception, MobileNetV2, and EfficientNetB0. After combining all the trained models, a diagnostic accuracy of 90.3%, sensitivity of 91.7%, and specificity of 89.4% were achieved.

On the other hand, the evaluation of the depth of myometrial invasion, typically using MRI, is an integral part of the assessment of patients suffering from endometrial cancer, as it affects the choice of treatment and prognosis. Therefore, AI-based MRI analysis appears as a possible time-efficient and cost-effective approach. Chen et al. evaluated the performance of a DL network in myometrial invasion depth identification on T2-weighted imaging (T2WI)-based endometrial cancer MRI [54]. Images from 530 patients with pathologically confirmed endometrial cancer were used to train and validate the model with a YOLOv3 algorithm to locate the lesion areas, achieving an accuracy of 84.8%, a sensitivity of 66.7%, and a specificity of 87.5% in determining deep myometrial invasion. When the performance of radiologists and trained network model were evaluated together, they reached a higher accuracy of 86.2% and a sensitivity of 77.8%, with equal specificity. In 2021, Zhu et al. developed a new method for the evaluation of depth of myometrial invasion MRI [55]. Differently from other previous prediction models, they used a geometric feature, named by the authors as LS, intended to describe the irregularity of the tissue structure inside the corpus uteri triggered by endometrial cancer. Then, a multiple probabilistic SVM incorporated LS and texture features, which are then merged to form the ensemble model EPSVM. The proposed EPSVM’s merging of LS and textural information showed more trustworthy predictions, achieving an accuracy, sensitivity, and specificity of 93.7%, 94.7%, and 93.3%, and exhibiting higher performance than those of the commonly used classifiers and the models using LS or texture features alone. Thus, future computer-aided classification based on the proposed method would be able to assist radiologists in accurately identifying deep miometrial invasion in MRI. On the other hand, the use of AI-models during the radiological diagnosis of endometrial cancer was also addressed in a few works. In 2021, Zhang et al. analyzed preoperative MRI from 158 patients with endometrial cancer and designed a CNN architecture to predict endometrial cancer based on radiomic features from MRI [56]. The AUC of the radiomic model was 0.897 in the training group. A comprehensive prediction model, incorporating specific imaging parameters and clinical pathological information, achieved an AUC of 0.913. Based on those results, the authors suggested that radiomics parameters can be used as noninvasive markers to predict endometrial cancer. In 2022, a Japanese group compared the diagnostic performance of a CNN model with the classification of three expert radiologists for diagnosing endometrial cancer [57]. The CNN demonstrated a non-inferior diagnostic performance than the radiologists. The single set of axial apparent diffusion coefficients of water maps and axial contrasted T1-weighted images revealed an AUC of 0.88–0.95. On the other hand, the addition of other image types had an associated AUC of 0.87–0.93.

The diagnosis of endometrial cancer is classically made after the analysis of histopathological material obtained during a hysteroscopy. Thus, AI may have a role in simplifying anatomopathological diagnosis, while reducing the problem of interobserver variability. Sun and colleagues built up a CAD approach based on a CNN and attention mechanisms, called HIENet, for histopathological endometrial images screening [58]. Their model was designed to discriminate between four classes of endometrial tissue, namely normal endometrium, endometrial polyp, endometrial hyperplasia, and endometrial adenocarcinoma. The ten-fold cross validation dataset revealed an accuracy of 76.9%, while the validation dataset of 200 hematoxylin and eosin images achieved an accuracy of 84.5%. By highlighting the histopathological correlations of local pixel-level image features to morphological characteristics of endometrial tissue, the model can assist pathologists in better interpretation of diagnoses.

Lastly, the value of ML and DL models is not only centered on predicting a diagnosis, but, more importantly, it provides significant prognostic information. In 2022, Feng et al. worked on a random forest (RF) model that was able to predict histology, stage, and grade of endometrial carcinoma preoperatively based on a database containing age, body mass index BMI, and examinations of 329 patients with endometrial cancer [59]. The RF model had an AUC of 0.69, accuracy of 81% for histology prediction, AUC of 0.66, and an accuracy of 63% for disease staging, with an AUC of 0.64 and accuracy of 43% for grading. The performance of doctors’ prediction compared to AI was higher than that of RF alone and doctors’ prediction without AI. Nevertheless, the modest results of the model need to be improved before its clinical implementation. More recently, Li et al. unveiled their work aimed at evaluating the performance of ML classification methods based on clinical and radiomic signatures from T2-weighted MR images in predicting deep myometrial infiltration, clinical risk category, histological type, and lymphovascular space invasion (LVSI) in women with endometrial cancer [60]. The AUCs for deep miometrial invasion, high-risk endometrial cancer, endometrial histological type, and LVSI classification were 0.79, 0.82, 0.91, and 0.85, respectively, on the independent external testing dataset. This work showcases the benefit of implementation of an ML model to obtain diagnostic and prognostic information during a single MRI exam.

### 2.3. Endometriosis

Endometriosis is a chronic medical condition, with a significant economic and disease burden on society [61,62]. It is defined as an extra-uterine growth of endometrial-like tissue in diverse organs, namely the ovaries, small bowel, colon, bladder, and peritoneum, causing pain and fertility issues. As a non-invasive and easily accessible tool, transvaginal ultrasound is commonly used in clinical practice for screening, but laparoscopic exploration with lesion sampling and histologic evaluation remains the gold standard approach for endometriosis diagnosis [63]. AI algorithms may play a key role in early detection of the disease, namely through automatic assessment of imagiology findings, which are usually difficult to interpret, or through the development of predictive models for earlier diagnosis and better disease control.

In fact, endometriosis consists of a myriad of symptoms, not rarely nonspecific, that complicate its diagnosis. In fact, the absence of clinical and minimally invasive markers of the disease result in a relevant number of diagnostic laparoscopies performed in this clinical context. In 2022, an ML algorithm based on 16 clinical and patient-based symptoms was developed [64]. Among the models tested, Soft Voting Classifier, random forest, and Extreme Gradient Boosting (XGBoost) stood out as those with the best performance, with a sensitivity and specificity ranging between 95%, 98%, and 80%, respectively. The high diagnostic yield suggests that the algorithm is a potential substitute for diagnostic laparoscopy, while also giving general care practitioners a possible tool for minimally invasive diagnosis or suspicion for this disease.

The current evidence suggests that endometriosis is characterized by a change in the amount of some molecules (i.e., proteins, antigens) in the blood, which can be evaluated using Raman spectroscopy, a non-invasive diagnostic method for endometriosis [65]. In 2019, a Turkish group report a Raman spectroscopy-based classification model developed from the blood samples of 94 patients (49 with endometriosis and 45 healthy individuals). Among the ML techniques tested, k Nearest Neighbors (kNN), achieved the best classification performance, with a sensitivity of 80.5% and a specificity of 89.7%. Once the model was tested with unseen data, it yielded a sensitivity and specificity value of 100%. This work suggested AI-based Raman spectroscopy classification as a potential future replacement for laparoscopy, given the minimally invasive nature of the exam, requiring only the collection of a peripheral blood sample.

In fact, colonic involvement in endometriosis is common and there are published works about the application of AI models in their diagnosis. An Italian group tested several ML models during the ultrasound (US) diagnosis of endometriosis with bowel involvement [66]. They compared the accuracy of different ML methods combining patient’s age with ultrasound soft markers, namely the presence of US signs of uterine adenomyosis, presence of an endometrioma, adhesions of the ovary to the uterus, presence of “kissing ovaries”, and absence of sliding sign, to raise suspicion of endometriotic bowel involvement. The models were developed based on data from 333 patients, with a testing dataset comprising 67% of the images, and a validation dataset with 33%. A Neural Network algorithm (NeuralNet) presented the best performance, with an accuracy of 73%, a sensitivity of 72%, a specificity of 73%, with a PPV of 52%, and an NPV of 86% for the diagnosis of rectosigmoid endometriosis. However, the model did not outperform current logistic regression models in terms of diagnostic accuracy, which limits its application in clinical practice.

Pouch of Douglas (POD) obliteration is a consequence of inflammation in the pelvis, often seen in patients with endometriosis. The sliding sign is a dynamic transvaginal ultrasound (TVUS) test that can diagnose POD obliteration. In 2021, a DL model was created based on a temporal residual network for automatic classification of the sliding sign as positive (normal) or negative (abnormal, indicating POD obliteration) using a dataset of 749 recorded ultrasound videos [67]. The model achieved an accuracy of 88.8%, with a sensitivity of 88.6%, a specificity of 90.0%, a PPV of 98.7%, and an NPV of 47.7% in the training dataset. However, despite the satisfactory performances of the model, there is a need to consider the technical difficulty of performing the ultrasonographic sliding sign, which could limit the generalization of the application of the DL model, and the absence of surgical information on POD, which nowadays remains the gold standard for its diagnosis.

In conclusion, several AI models (clinical, biochemical, and radiological) have been developed for an earlier, minimally invasive diagnosis of endometriosis. The main objective of this algorithms would be a reduction in the number of diagnostic laparoscopies performed in this context, which are commonly performed after months or years of disease symptoms and multiple exams with nondiagnostic findings. Nevertheless, all the algorithms were developed in a retrospective manner and need to be validated in prospective multicenter studies in order to replace the current gold standard and obtain an earlier diagnosis of this high-burden disease.

### 2.4. Ovarian Cancer

Initial characterization of a suspicious adnexal mass is based on imagological features from transvaginal ultrasonography and can be complemented using other effective tools, such as MRI or computed tomography (CT) [68]. Despite advances in therapy, ovarian cancer remains the most lethal gynecologic cancer, mainly because women are diagnosed at an advanced stage [69]. Therefore, improving the sensitivity of diagnostic tools, standardizing imaging techniques and developing predictive models for malignancy risk could reduce mortality from ovarian cancer by leading to the early detection of this malignancy [70].

Transvaginal ultrasound is commonly performed in the routine screening of ovarian cancer or following clinical suspicion in the presence of symptoms (namely abdominal pain, pelvic discomfort, or unexplained weight loss). Additionally, this exam can be performed preoperatively in the evaluation of an ovarian tumor. However, despite a satisfactory sensitivity for diagnosing ovarian cancer, its low PPV limits its implementation and results frequently in unnecessary procedures or concerns [71]. In fact, the distinction between benign and malign ovarian findings is challenging. In order to simplify this classification a SVM classification model to automatically discriminate malignant and benign ovarian tumors was developed and validated, using a dataset of 1000 benign and 1000 malignant ultrasound images [72]. They obtained an accuracy of 99.9%, a sensitivity of 100%, and a specificity of 99.8%.

Alqasemi and colleagues extracted twenty-four unique features from more than 400 ultrasound and photoacoustic images obtained from 33 ex vivo ovaries of 24 patients and used them to train three classifiers, namely generalized linear model, neural network, and SVM [73]. The main objective of the model was to differentiate between benign and malignant findings, with the SVM achieving the best results. At the validation dataset of unseen 95 images from 20 additional patients, the SVM classifier achieved 76.9% sensitivity and 95.1% specificity.

The automatic diagnosis of an ovarian tumor could also be based on variations of color intensity. Acharya et al. created a computer-aided diagnostic (CAD) technique called GyneScan^®^ for automatic ovarian tumor classification into benign or malignant, based on the subtle variations in the gray-level intensity variations in the 3D-transvaginal ultrasound images (1300 benign and 1300 malignant) [74]. K Nearest Neighbors/Probabilistic neural network classifiers with 11 classifiers showed 100% classification accuracy, sensitivity, specificity, and positive predictive value in detecting ovarian cancer. This research appointed the use of CAD during a transvaginal ultrasound as a valuable tool for increasing its diagnostic accuracy.

DL models have been appointed as a solution for increasing the diagnostic accuracy of a transvaginal ultrasound for ovarian cancer. A CNN based on 39 malignant and 105 benign cases was developed for automatic classification of adnexal masses, combining ultrasound images’ features and patient’s age [75]. The model revealed a global accuracy of 98.8%, sensitivity of 98.5%, and specificity of 98.9%. A CNN based on 3 DL algorithms (VGG16, ResNet50 and MobileNet) was developed and compared to the evaluation by an ultrasound expert [76]. The DL model showed comparable diagnostic accuracy with a sensitivity over 95% in the evaluation of 3077 ultrasound images from 758 women with ovarian cancer. The comparison with a radiologist expert was also addressed by Gao and colleagues. A retrospective dataset of 34,488 images of ovarian cancer and 541,442 images of benign findings was used to develop and validate the CNN in a multicenter setting [77]. The model presented higher accuracy when compared to radiologist assessment at detecting ovarian cancer (88.8% vs. 85.7%). These results are encouraging, given the specificity of transvaginal ultrasound, and AI-driven screening of ovarian cancer could be a realistic to achieve using nationwide screening even in unfavored settings. However, due to the retrospective nature of the studies, more investigations may contribute to the robustness of this experiment.

The diagnostic workup of an adnexal mass often includes CT imaging. A Chinese group developed a DL model to determine the risk of recurrence based on preoperative CT images from 245 patients with high-grade serous ovarian cancer [78]. The model incorporated DL features with a Cox proportional hazard model to automatically determine the 3-year recurrence probability. The combined model had an AUC of 0.772 and 0.825 for predicting 3-year recurrence in two validation cohorts. ML models were also developed based on contrast-enhanced CT images. An ensembled model with a combination of radiomics and DL features was developed for automatic discrimination of benign and malignant ovarian tumors [79]. The ML model showed a satisfactory performance, with an accuracy of 82%, specificity of 89% and sensitivity of 68%.

Additionally, artificial intelligence may have a role in augmenting the diagnostic accuracy of MRI. A dataset was composed of 55 sonographically indeterminate ovarian masses (27 benign and 28 malignant) [80], and, in this study, a prospective analysis of preoperative dynamic contrast-enhanced MRI was used to identify the best descriptive parameters in predicting malignancy of complex ovarian masses. Time-to-peak and wash-in-rate achieved the highest sensitivity and specificity. In the second part of the author’s experiment, and based on a combination of these two parameters, they developed a decision-tree classifier using the line equation obtained using linear discriminant analysis (LDA), which is a supervised ML classification model. The LDA model achieved an accuracy of 89% and AUC-ROC over 0.93. A retrospective study with 501 women intended to develop and validate an objective MRI-based ML assessment model to distinguish benign and malign epithelial ovarian tumors [81]. The ML performed better than radiologist assessment, with AUC values higher than 0.90. The importance of AI discrimination of adnexal masses is also the exclusion of the malignancy of an adnexal mass, reducing unnecessary surgeries and preserving ovarian function and fertility.

The application of AI models could also focus on discriminating ovarian cancer types, and not only in determining the malignant nature of an adnexal mass. A preliminary study by Zhang et al. evaluated the ability of an MRI radiomics model in discriminating benign ovarian diseases from malignant and differentiating between type I or II epithelial carcinomas [82]. For the classification between benign and malignant masses, the MRI radiomics model achieved a high accuracy of 87% in the independent validation cohort. For the classification between type I and type II subtypes, the method showed a satisfactory performance, presenting with an accuracy of 84% in the independent validation cohort.

On the other hand, there is a need to consider the use of AI models in the histopathological analysis of ovarian cancer. BenTaieb and colleagues developed an SVM model for automatic histopathological subtyping of ovarian cancer, based on a dataset of 133 patients [83]. Their model achieved substantial agreement with six clinicians that evaluated the same dataset, with a diagnostic accuracy of 90% in subtype discrimination. A Japanese group tried to predict the pathological result of an ovarian mass and evaluated the performance of five ML algorithms, namely support vector machine (SVM), random forest (RF), naive Bayes (NB), logistic regression (LR), and Extreme Gradient Boosting (XGBoost) in predicting the pathological diagnosis of ovarian tumors based on features, commonly available from blood tests, patient background, and data from preoperative examinations [84]. XGBoost was the one with better performance, with an accuracy of 80%.

Finally, AI may also play a role in giving accurate prognostic information for ovarian cancer patients. A British group focused on the development of a neural network capable of predicting the overall survival of epithelial ovarian cancer patients, comparing it with a logistic regression model [85]. The model outperformed the logistic regression model, predicting overall survival with an accuracy of 93%. When it came to predicting the outcome of surgery (complete/optimal cytoreduction vs. suboptimal cytoreduction), the neural network showed once more good results, with 77% accuracy. Late in 2022, a multicenter study aimed to develop an ML prediction model for the diagnosis and prognosis of epithelial ovarian cancer, based on age and 33 peripheral blood biomarkers from 521 patients with ovarian cancer and 144 patients with benign gynecological diseases [86]. XGBoost, a supervised ML method, showed promising results, as the AUC-ROC values distinguished epithelial ovarian cancer and benign findings, determining the pathological subtypes; grade and clinical stage were 0.958, 0.792, 0.819, and 0.68, respectively. The existence of validated models for preoperative prognosis information is important to assure the appropriate surgical treatment and select high-risk patients for monitoring recurrent disease, reducing ovarian cancer-related mortality.

### 2.5. Urogynecology

Urogynecology faces new challenges as we attempt to increase the diagnostic accuracy of different exams, while reducing interobserver variability [87]. Some studies have focused on the potential of AI methods in urogynecology as a diagnostic tool by boosting the capabilities of well-known techniques such as ultrasound, dynamic, and functional MRI, and standardizing urodynamic tests’ interpretation [88].

Stress incontinence is a highly prevalent condition associated with great morbidity. The disease is typically diagnosed using urodynamic tests, but other alternatives have been studied. A Taiwanese group developed a CAD system based on a multilayer perception neural network to diagnose stress incontinence based on anatomical and functional characteristics of the bladder neck on perineal ultrasound [89]. The proposed CAD system effectively detected USI using perineal sonographic analysis, with an accuracy of 91.7%, with a sensitivity of 94.4%, and a specificity of 83.3%. This study attests the ability of AI models to accurately identify imaging patterns. A few years latter a semiautomated pelvic floor measurement algorithmic model on 15 dynamic MRI was compared with manual pelvic floor measurements for pelvic organ prolapse evaluation [90]. The algorithmic model provided highly consistent and accurate locations for reference points on MRI, identifying them faster than the manual-point identification process. These results pave the way for research into new automatic methods to facilitate and improve the process of pelvic floor measurements on MRI based on the potential of artificial intelligence [89].

On the other hand, there have been a few studies evaluating the impact of AI models in the evaluation of urodynamic studies. Indeed, the application of AI algorithms could reduce the interobserver variability associated with exam interpretation. Detrusor overactivity, a marker of an overactive bladder, is detected in urodynamic studies and often correlates with lower urinary tract symptoms, driving management. In 2020, Wang et al. sought to develop a predictive model using ML algorithms to identify detrusor overactivity in urodynamic studies [91]. A total of 799 urodynamic studies were evaluated, and raw tracings of vesical pressure, abdominal pressure, detrusor pressure, infused volume, and all annotations during the exam were obtained. The ML model presented an overall accuracy of 81.3%, a sensitivity of 76.9%, and a specificity of 81.4% in detecting detrusor activity. A ML algorithm to detect detrusor overactivity in patients with spina bifida was developed using data windowing, dimensionality reduction, and SVM techniques [92]. In total, 805 urodynamic studies from 546 patients were used to train the model, which achieved a good performance in both time-based (AUC 0.919, sensitivity of 84.2% and specificity of 86.4%) and frequency-based (AUC 0.905, sensitivity of 68.3% and specificity of 92.9%) approaches. This promising proof-of-concept ML approach may be employed to standardize urodynamic studies’ interpretation and subsequently validate them as a useful tool in different populations.

Finally, there is also an interest in predicting responses to treatment in urogynecology, selecting the appropriated treatment for each patient. Sheyn et al. based on a retrospective dataset including 559 women with overactive bladders, who were treated with anticholinergic medications to develop and validate a predictive random forest model for anticholinergic response in this population [93]. Patients were stratified by age and number of previously failed medications. They achieved a final accuracy of 80.3%, with a sensitivity of 80.4% and a specificity of 77.4% in the external validation dataset. The model performed best in women aged younger than 40 years (AUC 0.84) and worst in women aged older than 60 years who had previously failed medication (AUC 0.71).

## 3. AI: Promises, Pitfalls, and the Unmet Needs for Its Implementation

AI-based systems have excelled in image analysis and interpretation and appeared throughout the last decade as powerful tools to revolutionize the field of gynecologic imaging. In the supra cited studies, AI was able to provide faster and accurate predictions and diagnosis, improving the overall efficiency of gynecologic healthcare. This is not a perspective in which these systems would replace clinicians, but instead they would perfectly integrate into clinical practice, assisting in the decision-making process and reducing classification errors and interobserver variability inherent to the human being, either by their erratic nature, or by the fatigue accumulated in healthcare professionals due to the ever-growing workload. In the field of gynecological cancer, undoubtedly one of the most promising aspects is the given capacity to analyze better and, especially, earlier, producing more reliable results and, ultimately, which may improve patient survival.

Beyond the convincing results of the mentioned experiments, most of these works were carried out using retrospective data analysis, so we cannot rule out selection bias or spectrum bias. Thus, these algorithms should be carefully tested before their implementation in daily practice. Other studies were performed with a small number of patients, thereby they still need to be validated using larger databases to attest their robustness. As AI tools themselves have the potential to improve their classification performance as new data are generated and they are fed with algorithms, the advent of the big data era will propel the exponential development of AI techniques in the near future. Improving the quality of input data collected in clinical practice, using standardized methods, is then a challenging requirement to ensure the increasing robustness of these techniques.

In fact, the application of AI-systems in gynecology is still in an embryonic phase in the imaging field. Indeed, there is a need to address the importance of data privacy and AI implementation bias. This novel healthcare technology is highly dependent on having a high amount of data, and its anonymization or re-identification is difficult and time-consuming, as is not always addressed [94,95]. The production of a large amount of information creates a problem in data management. The solution for this concern could be the generalization of blockchain technology in AI-produced data. A blockchain allows local storage of decentralized medical data, which remains immutable [96]. Thus, the implementation of blockchain technology in the next AI models is fundamental to assure the integration of ever-growing information.

On the other hand, it is important to address the problem of data bias. In fact, the development of AI models commonly has an inherent spectrum bias, in which the technology may not be applied to the population for which it was developed [95]. The majority of the works discussed in this review have a potential spectrum bias, as they were developed in a local or national patients’ dataset. Thus, the encouraging results of these models must be interpreted with caution, given the need to see the results validation in a heterogenous multicentric context, preferentially in a worldwide scenario, before implementing AI models into clinical practice.

Beyond the ever-evolving complexity in terms of model characteristics, there is also a concern in whether a model is trustable, and specially how it comes to a decision. Thus, several works have delved into the importance of explainable AI [97,98]. Thus, in order to be trustworthy, a ML or DL model should be capable of justifying the given output. Addressing this question is important to both the model developers and the regulatory entities, assuring accountability during the AI development process.

Furthermore, it is important to consider the implications associated with an AI-based decision. In fact, AI can produce good or bad outcomes, which can influence patient outcome [95]. Additionally, when facing an error in AI prediction, several factors must be considered, namely the quality of the model’s training, the type of algorithm and bias in data collection and analysis. However, patient safety should be a priority, and a model could be designed with the priority of greater sensitivity, even in the case of increasing false-positive cases. The matter of legal responsibility in AI-driven decisions has been the focus of recent papers, with a recent paper proposing the split of responsibility across three factors: the design of the model, the human–machine interaction, and the AI-driven human decision [99]. Indeed, commonly there will be difficulty in defining the point in the algorithm at which the decision was wrong, and clinicians must be able to coherently interpretate the model’s output, with the risk of reducing patient trust when facing errors caused by an AI-based decision [100]. Even so, currently there is an absence of well-defined regulations on ethical and legal issues with the use of AI in healthcare, and this topic should be a priority to standardize good practice with AI [101].

Additionally, the vast majority of the discussed works did not address the need for interoperability in AI-model implementation. In fact, the interoperability challenge is a hot topic when discussing the implementation of AI-systems in medicine. Indeed, in order to assure its clinical applicability, technology should be generalizable for the majority of devices available [102]. Despite showing promising results, the majority of AI systems evaluated in this review have demonstrated their results in a single hysteroscope, colposcope or even a single liquid-based cytology test. The inclusion of multidevice studies is fundamental to increase the technology readiness level of the different models.

Moreover, there is a need to consider the advent of generative AI and large language models in medicine, and, specifically, in gynecology. Large Language Models (LLMs) represent a category of DL technology developed to comprehend and produce language that closely mimics human, exemplified by entities like ChatGPT (OpenAI^TM^) or Google Bard (Google^TM^). These models are based on Transformer architectures, which use self-attention mechanisms, in Natural Language Processing (NLP), to identify complex relationships between words. In the medical field, transformative AI technologies such as these models could have a significant impact in clinical practice. They have the potential to facilitate the management of extensive electronic health records and large datasets, facilitating the resolution of complex clinical cases [103]. Additionally, they can contribute to advancements in machine translation (e.g., translating text to other languages) and enhancing the efficiency of the question–answer process (e.g., predict automatic answers based on the text at hand). The main limitation of using this type of technology is its propensity to introduce errors, as generated text may appear trustworthy despite being factually incorrect (hallucination effect) [104]. These chatbots often prioritize following instructions rather than providing genuine responses, lacking the authentic approach that a human would offer [105]. The referencing process also lacks proper control, potentially resulting in mistakes. Additionally, the unpaid technology of ChatGPT (ChatGPT 3.5) has not been upgraded, with the model not incorporating the latest information beyond 2021 into its training data. In terms of the commercial version, the paid version, ChatGPT 4, outperforms the prior free version, ChatGPT 3.5 [106]. This enhancement has the potential to reduce medical errors and decrease fatigue due to its enhanced processing capability, which includes the ability to handle pictures and more complex data. Such developments might be extremely useful for streamlining information flowcharts, improving doctor–patient communication, and minimizing technical errors. Thus, while LLMs may introduce bias or incorrect information, they can be very useful in the medical context, particularly for summarizing vast amounts of information. This becomes especially valuable in an era where medical knowledge is growing exponentially, with genecology being a suitable area for NLP models [107]. However, before implementation and generalization, regulation of and compliance with ethical issues should be assured to augment the clinical utility of the models.

## 4. Concluding Remarks

The ever-growing development of AI technologies and their increasing potential in numerous areas of healthcare make this a trending topic. Apart from several challenges facing its clinical implementation, the future seems to be very promising in gynecology since some interesting advances have been made. Undoubtedly, these auxiliary computerized methods proved to be profitable and time- and resource-saving. However, more research studies are needed to attest the usefulness of this technology in real life. The developments until this moment have been tremendous, and even more are expected over the next few years. In fact, there is still a very long way to go until AI-based technologies become perfectly integrated into everyday clinical decisions.

## Figures and Tables

**Table 1:** Summary of machine and deep learning models that have been focused on in this review, with a brief consideration of their methodological concepts. ML—machine learning; DL—deep learning; CNN—convolutional neural network.

Type of Model	Model	Definition
ML	Support Vector Machine (SVM)	Supervised algorithm that identifies an optimal hyperplane to classify data into distint categories defining the optimal margin between the categories.
ML	PNN (Probabilistic Neural Network)	Non-parametric neural network capable of pattern recognition and classifcation, estimating probability through a Parzen window aproach.
ML	Fuzzy C-Means	Algorithm that classifies all data into multiple clusters (differently to most of the models that atribute a classification to a single category), being specially useful in cases where data or images may be partially atributable to more than one category.
ML	Random Forest	ML algorithm that constructs multiple decision trees, combining them to develop an accurate model for classification and regression tasks, reducing overfitting in complex datasets has each tree and has its individual prediction, combining them in the final development of the model.
ML	XGBoost	Gradient boosting algorithm that sequentially builds an ensemble of individual decision trees, and it is used in classification and regression tasks.
ML	kNN (k-Nearest Neighbors)	ML model that makes predictions using a non-parametric method, based on the majority class or average value of the k value nearest the data points.
DL	ResNET (Residual Network	DL architecture that introduces residual connections, faciliting the learning and updating of residual mapping while reducing the vanishing gradient method, being one of the most used DL models in the classification of image patterns.
DL	U-NET	CNN designed for image segmentation tasks with multiple convolutional layers, capturing information effectively while mantaning spacial detail, and it is helpful in assuring lesion location and pixel-level accuracy.
DL	MASK-R	DL architecture suitable for segmentation of images, and which is capable of identifiying frames with the relevant lesion while assuring the lesion is location inside the image.
DL	YOLO (You Only Look Once)	DL algorithm that processes images in a single pass, making all predictions at once, which facilitates its real-time application.
DL	EB-0 GRU	Hybrid approach combining both a CNN (E-B0) with a recurrent neural network with gated units (GRU), facciliting image detection and segmentation.
DL	ShuffleNet	CNN architecture for image classification, with a design that reduces the computacional complexicity, lowering the computational requirements.
DL	VGG-Net	Neural network composed of small 3 × 3 convolutional filters creating a uniform deep structure, which allows improved accuracy in image detection/recognition.
DL	MobileNetV2	Neural architecture designed for mobile devices, with lightweight concolutions, and which is effective with lower computational requirements.

**Table 3:** Summary of studies about AI implementation in hysteroscopy. Sn, sensitivity; Sp, specificity; AUC—area under the curve; NK—not known; EH—endometrial hyperplasia; AH—atypical hyperplasia; EC—endometrial cancer; EP—endometrial polyps; SM—submucous myomas; FCM—Fuzzy C-Means.

Author, Year	Study Aim	Pati-ents *n*	Frames *n*	Pathologic Confirma-tion	AI Method	Dataset Method	Analysis Method	Catego-Ries	Performance Metrics %		
									Sn	Sp	AUC
Neofytou, M.S.; 2006, USA [50]	Hysteroscopy image classification	198	418 frames – Normal: 209 – Abnormal: 209	No	Color– texture analysis methods	Frame annotation based on texture features (two different classifiers)	10-fold cross validation (and leave-one-out method)	Normal vs. abnormal	51–77	72–82	NK
Vlacho-kosta, 2013, Greece [51]	Differentiating normal vs. uterine vs. endometrial cancer	77	NK Only number of patients per category	Yes	DNN and FCM	Feature extraction related to vessel and texture structure	NK	Normal vs. patological	71–93	71–91	91
Zhang, 2021, China [52]	Differentiating benign (EH, EP, and SM) from premalignant/malignant lesions (AH and EC)	454	1851 frames: EH = 509 AH = 222, EC = 280 EP = 615 SM = 225	Yes	VGGNet	Image-based frame labeling with preprocessing and retaining of region of interest and data augmentation	Train–test validation (50 images for each category in test set)	Part 1: EH vs. AH vs. EC vs. EP vs. SM; Part 2: benign vs. premalignant/malignant	83	96	94
Takahashi, 2021, Japan [53]	Differentiating malign vs. benign or normal findings	177	411,800 frames – Malignant: 109,957 – Others: 301,843	Yes	Xception, MobileNetV2 and EfficientNetB0	Frame labeling in still images and video segments	Train–testvalidation	Malignant and others (uterine myoma, EP normal endometrium)	92	89	90

## Data Availability

The authors will provide additional information on their research. For more information, please contact the corresponding author.
